# EczemaPred: A computational framework for personalised prediction of eczema severity dynamics

**DOI:** 10.1002/clt2.12140

**Published:** 2022-03-28

**Authors:** Guillem Hurault, Jean François Stalder, Sophie Mery, Alain Delarue, Markéta Saint Aroman, Gwendal Josse, Reiko J. Tanaka

**Affiliations:** ^1^ Department of Bioengineering Imperial College London London UK; ^2^ Clinique Dermatologique University Hospital Nantes France; ^3^ Pierre Fabre Laboratories Toulouse France

**Keywords:** atopic dermatitis, Bayesian model, machine learning, PO‐SCORAD, prediction

## Abstract

**Background:**

Atopic dermatitis (AD) is a chronic inflammatory skin disease leading to substantial quality of life impairment with heterogeneous treatment responses. People with AD would benefit from personalised treatment strategies, whose design requires predicting how AD severity evolves for each individual.

**Objective:**

This study aims to develop a computational framework for personalised prediction of AD severity dynamics.

**Methods:**

We introduced EczemaPred, a computational framework to predict patient‐dependent dynamic evolution of AD severity using Bayesian state‐space models that describe latent dynamics of AD severity items and how they are measured. We used EczemaPred to predict the dynamic evolution of validated patient‐oriented scoring atopic dermatitis (PO‐SCORAD) by combining predictions from the models for the nine severity items of PO‐SCORAD (six intensity signs, extent of eczema, and two subjective symptoms). We validated this approach using longitudinal data from two independent studies: a published clinical study in which PO‐SCORAD was measured twice weekly for 347 AD patients over 17 weeks, and another one in which PO‐SCORAD was recorded daily by 16 AD patients for 12 weeks.

**Results:**

EczemaPred achieved good performance for personalised predictions of PO‐SCORAD and its severity items daily to weekly. EczemaPred outperformed standard time‐series forecasting models such as a mixed effect autoregressive model. The uncertainty in predicting PO‐SCORAD was mainly attributed to that in predicting intensity signs (75% of the overall uncertainty).

**Conclusions:**

EczemaPred serves as a computational framework to make a personalised prediction of AD severity dynamics relevant to clinical practice. EczemaPred is available as an R package.

## 1 INTRODUCTION

Atopic dermatitis (AD or eczema) is a common chronic inflammatory skin disease characterized by dry and itchy skin.^1^ Disease symptoms manifest as relapses and remissions that are often unpredictable, making treatment difficult, and increasing patients' burden. Current treatments for mild and moderate AD are an application of emollients on dry skin and anti‐inflammatory creams or ointments (topical corticosteroids and calcineurin inhibitors) on inflammatory skin. Tailoring treatment strategies to each patient's conditions is essential to achieve maximum effectiveness because treatment responses often differ between and within patients.^2^, ^3^

Designing personalised treatment strategies requires predicting future disease states for individual patients as AD symptoms fluctuate dynamically in a highly heterogeneous manner. We have recently demonstrated that it is possible to predict the patient‐specific daily evolution of AD severity by developing a mechanistic Bayesian machine learning model.^4^ The model captured the patient‐specific heterogeneity in dynamic trajectories of AD severity and responsiveness to treatment. However, its predictive performance and clinical applicability were limited because the model was developed using a daily bother score, which is a subjective global measure of distress caused by AD and is not suitable to capture different aspects of AD symptoms reliably. Using a validated objective severity score that combines multiple severity items could improve the predictive performance and make predictive models more relevant for clinical practice.

The Harmonising Outcome Measures for Eczema (HOME) initiative recommended the Eczema Area and Severity Index^5^ (EASI) as the core outcome instrument for clinical signs of eczema to be measured in clinical trials.^6^ SCORing AD^7^ (SCORAD) and its objective component (oSCORAD) have also been validated as outcome instruments,^8^ and other scores such as Six Area Six Signs AD^9^ (SASSAD) are still routinely used in clinical practice. All these instruments report AD severity as a single score obtained by aggregating the severity scores for multiple severity items, including intensity signs, subjective symptoms and extent (the area affected by eczema). Each severity item captures a different aspect of AD severity and may follow its own dynamics.

In this paper, we introduce EczemaPred, a computational framework to predict patient‐specific dynamic evolution of AD severity. It is based on the idea that modelling the evolution of each relevant severity item and aggregating the predictions could improve the performance and the clinical relevance of the prediction of AD severity dynamics. EczemaPred consists of a collection of Bayesian state‐space models that describe the item‐dependent dynamics of each severity item. The predictions for any AD severity score can be obtained by aggregating the predictions for the relevant severity items made by their Bayesian state‐space models.

We use EczemaPred to predict patient‐dependent dynamic evolution of the Patient‐Oriented SCORAD (PO‐SCORAD),^10^ a validated self‐assessment of SCORAD^11^ that can be recorded on a smartphone app. Self‐assessments of AD severity are more suitable for tracking the short‐term (daily to weekly) evolution of severity dynamics than clinical assessments that can be performed only during clinical consultations of a limited frequency. PO‐SCORAD is one of the core instruments recommended by the HOME initiative to measure patient‐reported symptoms in clinical practice.^12^ We validate the EczemaPred approach using longitudinal datasets from two clinical studies, a dataset from a published study^13^ and another dataset we collected in an observational study.

## 2 METHODS

### 2.1 Observational study for data collection

An observational study (ClinicalTrials.gov, NCT04553224) was conducted from November 2019 to February 2020 in Toulouse (France) following the approval by IEC (CPP Ile de France V, Saint Antoine Hospital, *n*°582,211). We recruited 16 adult AD patients (mean age 25 years old, SD = 5) whose SCORAD were between 20 and 40 (mean SCORAD 34.6, SD = 4.4 at inclusion). Patients recorded PO‐SCORAD using an app (https://www.poscorad.com) for up to 12 weeks every day while continuing their usual treatment. In the case of AD flare (*n* = 8 patients), medication was changed by the investigators. Informed consent was obtained from all study participants.

### 2.2 Datasets used for predictive modelling

We used two datasets with daily to weekly measurements of PO‐SCORAD and its severity items over a moderately long period. The first dataset, referred to as dataset 1, is from a published study investigating the role of an emollient in children (mean age 3.6 y.o., SD = 1.3) with mild to moderate AD.^13^ Dataset 1 consists of PO‐SCORAD recorded for 347 children approximately twice weekly (usually every 3 or 4 days) for up to 17 weeks (119 days), resulting in 9943 observations. 11 children with less than five observations in the original study were excluded. The second dataset, referred to as dataset 2, was obtained from the observational study described in the previous subsection. The data consists of PO‐SCORAD recorded by 16 adult AD patients daily for up to 12 weeks (84 days), resulting in 1136 patient‐day observations and 13.6% missing values.

**FIGURE 1:**
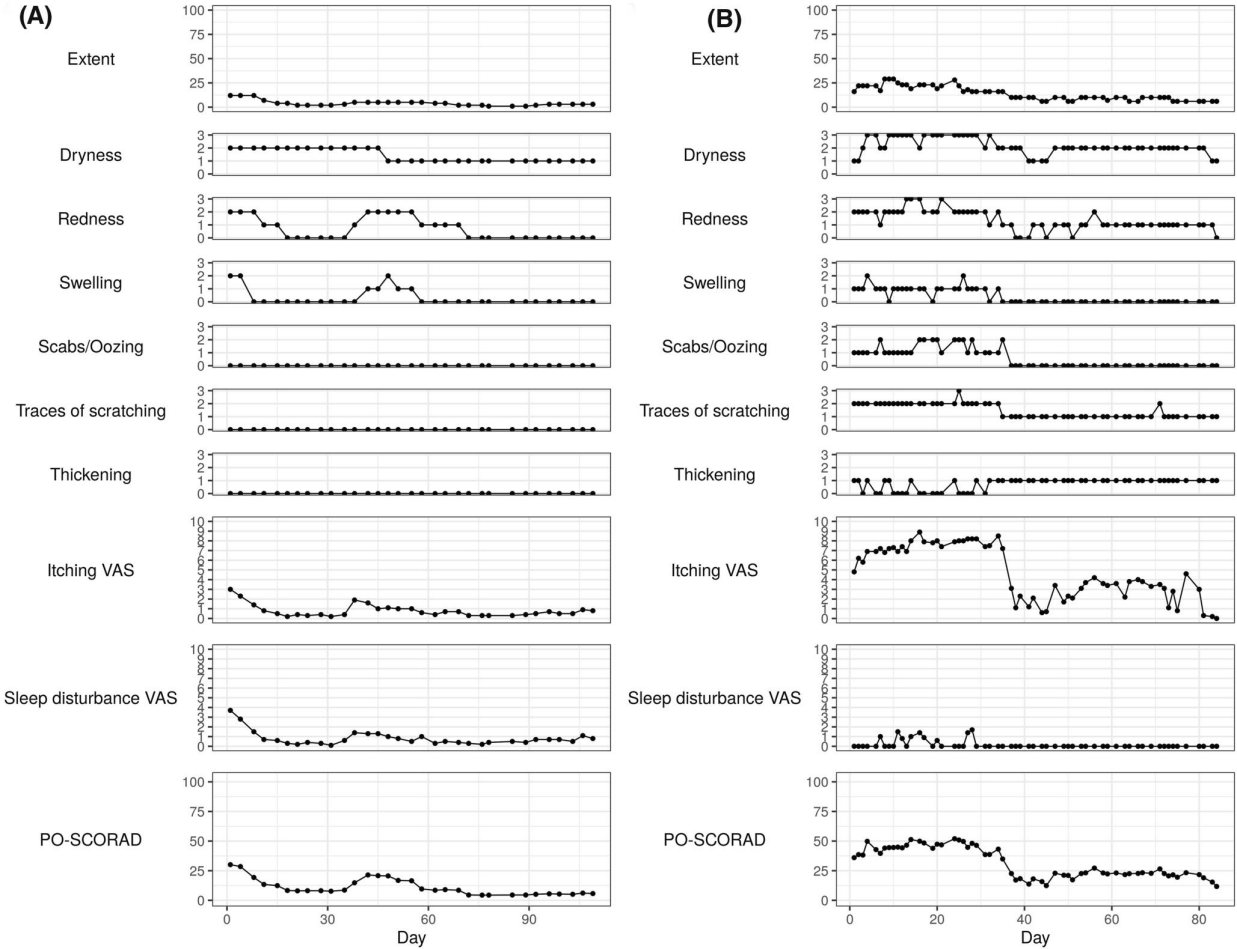
Example trajectories of PO‐SCORAD and its severity items for representative patients from datasets 1 (A) and 2 (B)

Dataset 1 had 70.3% missing values if it was expected to have daily recordings. Compared to dataset 2, dataset 1 had more missing values due to less frequent recordings (3 to 4 times fewer observations) per patient but contained about nine times more observations in total, as it was collected from 21 times more patients (Table 1). The severity dynamics appeared to be relatively more stable in dataset 1 than in dataset 2 (Figure 1).

**TABLE 1:** Characteristics of datasets

	Dataset 1	Dataset 2
Number of subjects	347	16
Age (mean ± SD)	3.6±1 0.3	24.7±5 0.0
PO‐SCORAD recording	Twice weekly	Daily
Duration	Up to 17 weeks	Up to 12 weeks
Missing values for daily recording	70.3%	13.6%
Observations	9943	1136
PO‐SCORAD at inclusion	31.2±7.7	34.6±4.4
Data collection	Subject notebook	Smartphone app

PO‐SCORAD is defined by 0.2A+3.5B+C, where A∈[0,100] corresponds to the extent (the percentage of the area affected by eczema in the whole body), B∈[0,18] to intensity signs and C∈[0,20] to subjective symptoms. The intensity signs component (B) is the sum of the scores for six intensity signs (dryness, redness, swelling, oozing, scratching and thickening), each of which is assessed on a representative area for that sign (an area where the sign has an ‘average’ intensity) using an ordinal scale as 0 (absent), 1 (mild), 2 (moderate) or 3 (severe). The subjective symptoms component (C) is the sum of scores for two symptoms (itching and sleep loss), each of which is assessed on a visual analogue scale from 0 (no symptom) to 10 (severe symptom). In both datasets 1 and 2, the extent (A) takes discrete values (0, 1, …, 100 with a resolution of 1), and each subjective symptom score takes discrete values (0.0, 0.1, …, 10.0 with a resolution of 0.1). The objective component of PO‐SCORAD (PO‐oSCORAD) is calculated by 0.2A+3.5B.

We did not use demographic or treatment information in our models because our previous study^4^ suggested that their inclusion does not show a noticeable improvement in the predictive performance for patient‐specific daily evolution of AD severity. In this study, we aimed to develop simple models with a good predictive performance that could be extended later to investigate the effects of demographics or treatment.

### 2.3 Model overview

**FIGURE 2:**
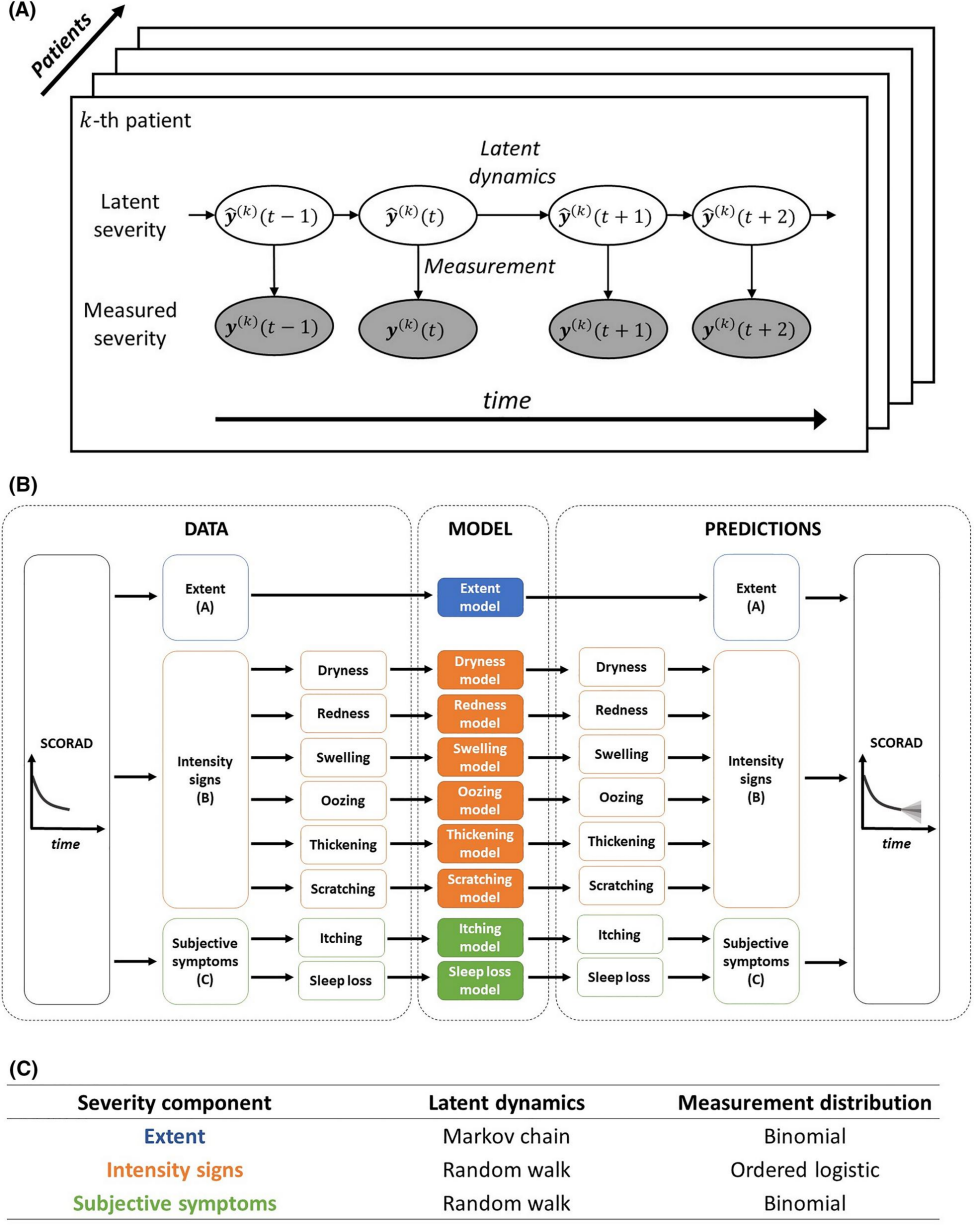
Model overview. (A) Bayesian state‐space models in EczemaPred. Each model describes the dynamics of a latent severity (white ovals) and the measurement of the latent severity to obtain the recorded severity (grey ovals). (B) Use of EczemaPred for SCORAD prediction. Predictions from nine models (coloured rectangles), each of which corresponds to one of the nine severity items for SCORAD, are aggregated to provide predictions for SCORAD. (C) Latent dynamics and measurement distributions for the three severity components of SCORAD

We introduce EczemaPred, a collection of machine learning models (Bayesian state‐space models) that can be used to describe the data‐generating mechanisms of each severity item. Each model assumes the existence of a true latent (unobserved) severity that follows its own latent dynamics and that the recorded severity was obtained as a result of imperfect measurement of the latent severity (Figure 2A).

EczemaPred to predict SCORAD (and PO‐SCORAD as its self‐assessment version) consists of nine models, each corresponding to one of the nine severity items for SCORAD (Figure 2B). Predictions from the nine models are aggregated to produce predictions for SCORAD by assuming independence of the severity items. The latent dynamics and measurement distributions of the state‐space models were tailored to each severity item (Figure 2C).

We modelled the latent dynamics for the extent as a Markov chain that describes how a small “patch” of the skin transitions from non‐lesional to lesional and vice versa. A binomial distribution was used to count the numbers of lesional patches, that is the extent. We assumed random walk latent dynamics for the intensity signs and subjective symptoms in the absence of precise insights about the item‐specific data‐generating mechanisms. The measurement of intensity signs from the latent scores was described by an ordered logistic measurement distribution and that of subjective symptoms by a binomial distribution.

Parameters of the measurement distributions and the variance of the random walk latent dynamics were shared between patients, while parameters of the Markov chain latent dynamics were made patient‐dependent with hierarchical priors. Priors were chosen to be weakly informative. Missing values were treated as an absence of measurement in the state‐space models. Details of the models, parametrisations and choice of priors are described in Supplementary A.

Model inference was performed using the Hamiltonian Monte Carlo algorithm in the probabilistic programming language Stan^14^ with four chains and 2000 iterations per chain, including 50% burn‐in. Prior predictive checks and fake data checks were conducted.

### 2.4 Model validation

We evaluated the predictive performance of our models in a forward‐chaining setting (Figure S3), where we trained the models every 4 days. That is, we first trained the models on the first day's data of a patient and tested them using their data over the next 4 days, then trained the models on the first 5 days' data and tested them on the next 4 days' data, etc.

The probabilistic predictions of PO‐(o)SCORAD and its nine severity items were evaluated using a logarithmic scoring rule, the log predictive density (lpd).^15^ We also computed an accuracy metric for PO‐(o)SCORAD predictions, defined as the probability that the predictions were within five units of the measured score. We plotted the lpd and the accuracy as a function of the number of training observations (equivalently the number of training days) to produce learning curves. Details of the performance metrics are given in Supplementary B.

We compared the predictive performance of EczemaPred with that of reference models, including a uniform forecast, a historical forecast, and a random walk model (which provides a flat forecast, i.e. centred on the last observed value). We used Markov chain models instead of random walk models as references for the six intensity signs that take discrete values in [0, 3]. For PO‐(o)SCORAD predictions, we also compared the performance of EczemaPred to that of standard time‐series forecasting models, including an exponential smoothing model, an autoregressive model and a mixed effect autoregressive model. Details of the reference models are given in Supplementary C.

## 3 RESULTS

All EczemaPred models and reference models were fitted successfully for all severity items on the two datasets. We found no evidence of an absence of convergence by monitoring trace plots and by checking the potential scale reduction factor ($\hat{R})$.^16^ We conducted posterior predictive checks and found no clear discrepancies between the data and the models' simulations.

### 3.1 Predictions of severity items

**FIGURE 3:**
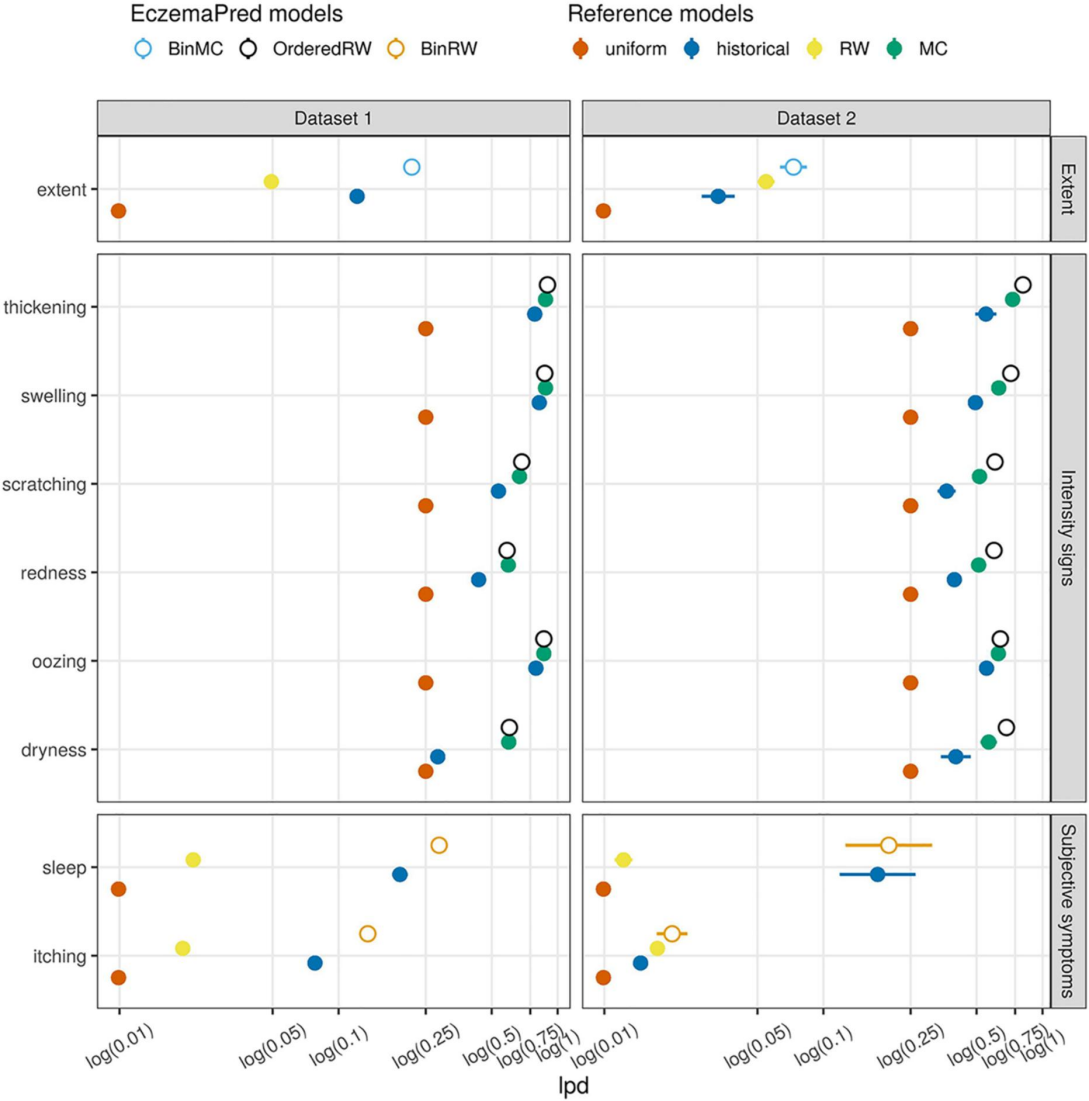
Predictive performance for 4‐day‐ahead forecasts by EczemaPred models (empty circles) and reference models (filled circles) measured by lpd (the higher, the better). EczemaPred models are a binomial Markov chain model (BinMC) for extent, an ordered logistic random walk model (OrderedRW) for intensity signs, and a binomial random walk model (BinRW) for subjective symptoms. Reference models include a uniform forecast (uniform), a historical forecast (historical), a random walk model (RW), and a Markov chain model (MC). The performance was calculated after training with approximately 80% of the data (77 days' data for dataset 1 and 65 days' data for dataset 2)

EczemaPred models learned the dynamics of severity items as more data came in (Figures S4–S12, top). A similar predictive performance was confirmed for the models trained with dataset 1 and those with dataset 2, supporting the generalisability of the models. However, predictions of extent and itching appeared to be more difficult with dataset 2 than with dataset 1 (Figure 3, S4 and S11). For example, the lpd for predicting extent is much higher for the EczemaPred model trained with dataset 1 than with dataset 2 (−1.53±0.07 vs. −2.62±0.14) after training with 80% of the data.

EczemaPred models outperformed the reference models for the two datasets in terms of predictive performance (Figure 3 and S4–S12, top). EczemaPred models showed only marginally better predictive performance than the historical forecasts for thickening, swelling and oozing. The lpd of the historical forecast was already close to the maximum lpd of 0 for those intensity signs that had a low prevalence (Figures S1–S2) and were easier to predict than other signs. A historical forecast tended to outperform a random walk model for extent and subjective symptoms that do not demonstrate persistent dynamics. For intensity signs whose dynamics are often persistent, a Markov chain model performed almost as well as the EczemaPred model with an ordinal logistic measurement and latent random walk.

The predictive performance decreased as the prediction horizon increased for all the models investigated. The decrease in lpd when the prediction horizon is increased by a day was similar or smaller for EczemaPred models compared to the reference models with a non‐constant forecast (Figures S4–S12, bottom).

### 3.2 Predictions of PO‐(o)SCORAD

**FIGURE 4:**
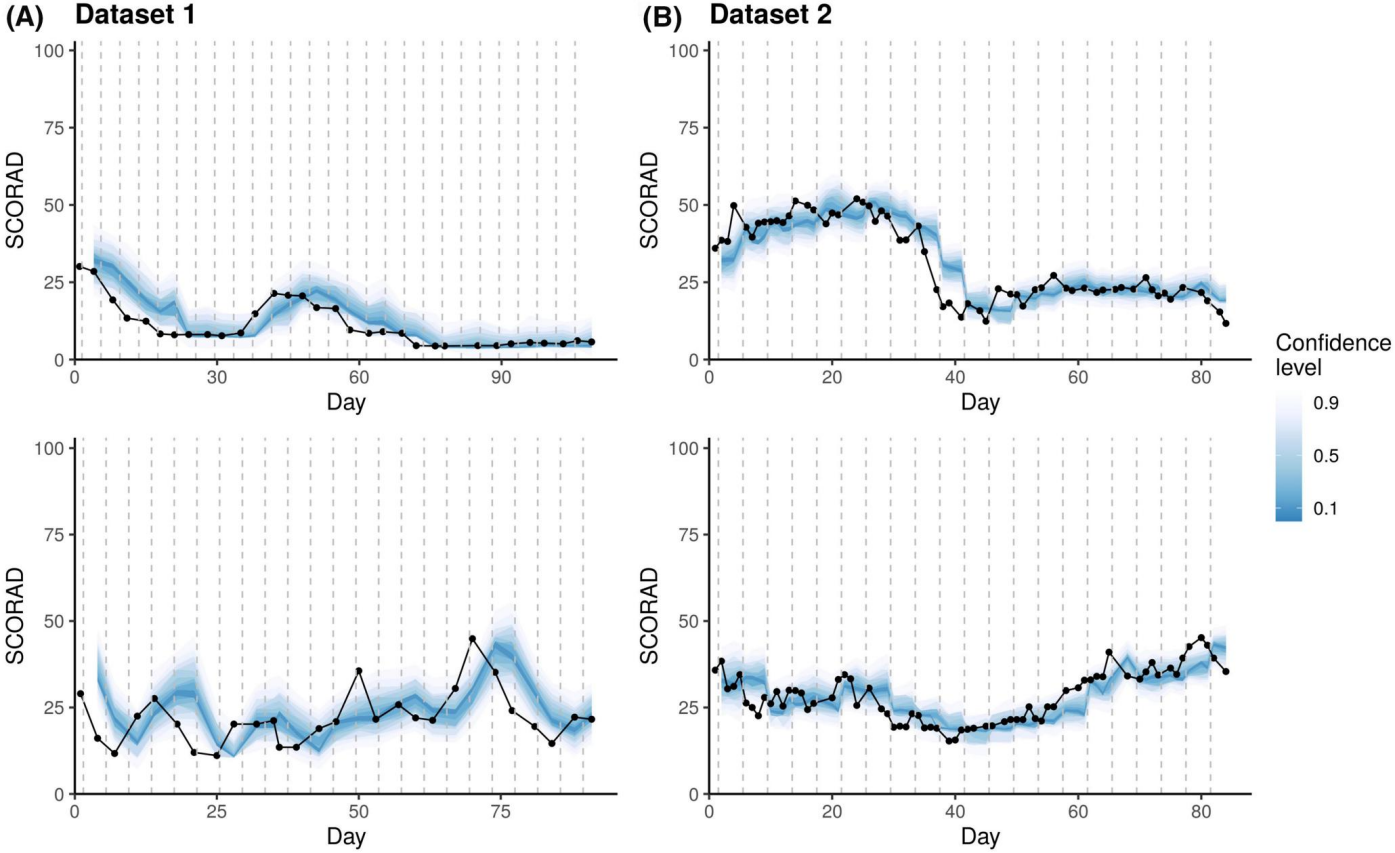
PO‐SCORAD prediction by EczemaPred for four representative patients from dataset 1 (A) and dataset 2 (B). Coloured ribbons correspond to stacked prediction intervals of highest density (darkest ribbon corresponds to the mode), and black dots represent the recorded PO‐SCORAD. The model is updated, and new predictions are issued every 4 days (vertical dashed lines)

Predictions for PO‐SCORAD were derived by aggregating the predictions of the severity items by their respective models (example predictive trajectories in Figure 4).

**FIGURE 5:**
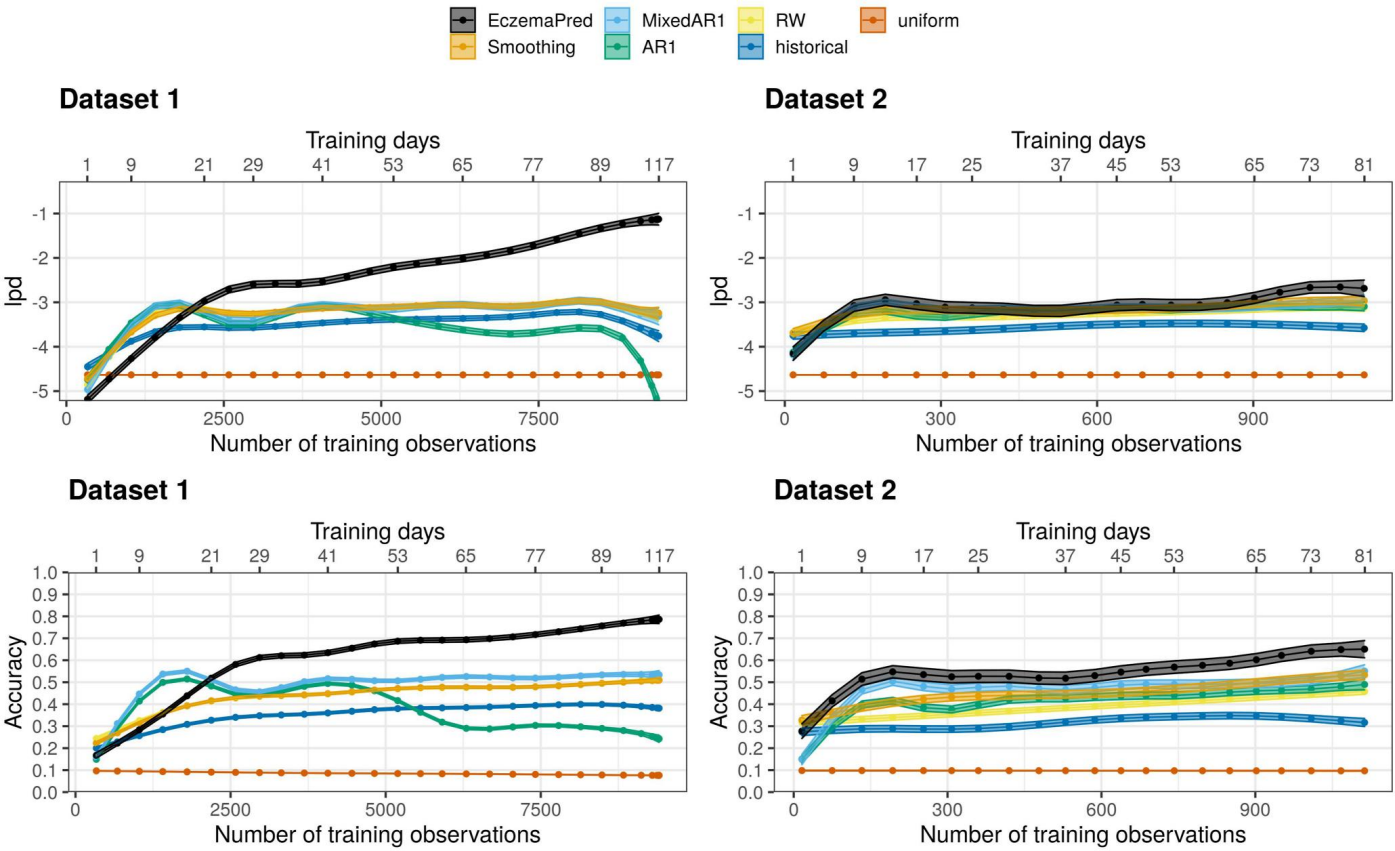
Learning curves for 4‐day‐ahead forecasts of PO‐SCORAD evaluated by lpd (top) and accuracy (bottom) as a function of the number of training observations (training days), for datasets 1 (left) and 2 (right). EczemaPred models perform better than reference models, including an exponential smoothing model (Smoothing), a mixed effect autoregressive model (MixedAR), an autoregressive model (AR), a random walk model (RW), a historical forecast (historical), and a uniform forecast (uniform)

We confirmed that the performance of PO‐SCORAD prediction by EczemaPred improved as more data came in but did not plateau (Figure 5). It suggests a possibility of further improvement of the performance if more training data was available and a need for more accurate estimation of some model parameters. In contrast, the performance of the reference models stopped improving, suggesting that the improvement observed for EczemaPred was not due to a change in the data distribution (e.g. due to patients dropping the study early).

EczemaPred outperformed the reference models that predict PO‐SCORAD directly (rather than aggregating the prediction of severity items as in EczemaPred), supporting our approach. The difference in lpd between EczemaPred and the reference models is less evident in dataset 2 than in dataset 1 for PO‐SCORAD prediction. The difference is more evident for PO‐oSCORAD prediction (Figure S13) due to the lower predictive performance for subjective symptoms with dataset 2 than dataset 1. Otherwise, similar results with comparatively better predictions were obtained for PO‐oSCORAD.

The exponential smoothing and the (mixed) autoregressive models achieved similar predictive performance to the random walk model (Figure 5) as they tend to emulate a random walk behaviour. The exponential smoothing model has a smoothing factor of 1, and the autoregressive models have an autocorrelation parameter of 1 and an intercept of 0. The fact that complex models emulate a simpler random walk model highlights the difficulty of developing accurate predictive models using only the aggregate PO‐SCORAD data.

The learning curves (Figure 5) indicate that EczemaPred achieved the accuracy of 71.8±1.0% (mean ± standard error) after training with 77 days' data from dataset 1 (60.2±2.8% with 65 days' data from dataset 2). That is, the 4‐day‐ahead prediction by EczemaPred is within five units of the measured PO‐SCORAD with a 71.8% probability for an average patient. The accuracy values of the reference models were much lower, with 39.4±0.5% for the historical forecast with dataset 1 (34.9±1.4% with dataset 2), 47.9±0.5% (43.1±1.2%) for the random walk model, and 51.9±0.7% (49.5±1.9%) for the mixed effect autoregressive model. The improvement in the accuracy from the random walk model to EczemaPred (+23.9%, +17.1%) is larger than that from the historical to the random walk model (+8.5%, +8.2%), although ‘the marginal gain from complicated models is typically small compared to the predictive power of the simple models’.^17^

The predictive performance of EczemaPred with dataset 1 appeared to be better than that with dataset 2 (Figure 5), although the actual predicted dynamics do not always appear qualitatively different between the two datasets (Figure 4). Several data characteristics (e.g. dataset size, frequency of measurements and demographics) may explain the difference, but it is difficult to pinpoint the main factors without a meta‐analysis. It is also possible that the performance with dataset 2 becomes comparable or superior to that with dataset 1 if we allow for a more prolonged training phase, given that the performance did not plateau.

The predictive performance of EczemaPred and the reference models decreased as the prediction horizon increased (Figure S14), similarly to what was observed for individual severity items. The accuracy of EczemaPred was estimated to decrease by approximately 3.0±0.2% on average when the prediction horizon was increased by 1 day in dataset 1 (3.9±0.5% in dataset 2). It leads to an accuracy of 80.7±1.2% and 62.8±1.1% for one‐day‐ahead and one‐week‐ahead forecast, respectively, for dataset 1 (71.9±2.9% and 48.6±3.4% for dataset 2). These results suggest that EczemaPred performance would become equivalent to a historical forecast for 15.2 days‐ahead predictions and 10.4 days‐ahead predictions for datasets 1 and 2, respectively, assuming that the extrapolation of accuracy loss is valid. A similar decrease in accuracy was estimated for the reference models.

### 3.3 Decomposition of prediction uncertainty in EczemaPred

We investigated which of the three components of PO‐SCORAD (0.2A,3.5B or C) contributed to the uncertainty in PO‐SCORAD prediction the most by computing the proportion of the variance of each component to the variance of the PO‐SCORAD for each prediction. On average, 7% of the uncertainty in PO‐SCORAD prediction could be attributed to the extent (0.2A), 79% to the intensity signs (3.5B) and 14% to the subjective symptoms components (C) for dataset 1 (5%, 72% and 23% for dataset 2). In contrast, the intensity signs component is $\frac{63}{103}\approx 61\text{\%}$ of the total SCORAD, with extent and subjective symptoms each contributing to $\frac{20}{103}\approx 19\text{\%}$. Accordingly, improving predictions of intensity signs is the most promising option to improve predictions of PO‐SCORAD. The intensity signs that contribute to the prediction uncertainty the most were calculated to be dryness, redness and scratching, the other signs being less prevalent.

## 4 DISCUSSION

This paper introduced EczemaPred, a computational framework to predict the patient‐dependent dynamic evolution of AD severity using machine learning (Figure 2). We validated EczemaPred in predicting PO‐SCORAD using two independent datasets with different characteristics.

EczemaPred for predicting PO‐SCORAD consists of nine Bayesian state‐space models, one for each severity item of PO‐SCORAD (extent, six intensity signs, and two subjective symptoms). EczemaPred models outperformed the reference models we considered for all the severity items (Figure 3). Predictions of PO‐SCORAD were produced by aggregating the predictions by the severity item models and outperformed those obtained by standard time‐series forecasting models (Figure 5). The prediction accuracy was approximately 72% and 60% for 4‐day‐ahead forecasts for datasets 1 and 2, respectively. Most of the prediction uncertainty in PO‐SCORAD (79% and 72% for datasets 1 and 2, respectively) could be attributed to the intensity signs component, suggesting that improving predictions of intensity signs is the most promising approach to improve PO‐SCORAD predictions.

Modelling the dynamics of each severity item has several advantages when the breakdown of the aggregate severity score is available. It enables us to extract more signals from the data, as the AD severity dynamics for each patient are described by multiple time‐series, one for each severity item (nine using PO‐SCORAD) instead of one for the aggregate score. This approach also reduces the uncertainty in the aggregate score prediction when some severity items are easier to predict than others (e.g. when they are not very prevalent or do not vary much over time). The models can be tailored to each severity item to reflect the item‐dependent data‐generating mechanisms with relevant measurement processes and latent dynamics. The models are thus more interpretable and transparent, as predictions of aggregate severity scores can be decomposed into predictions of their components.^18^ The models could be used to predict any combination of the severity items (e.g. PO‐oSCORAD) without potential inconsistencies in predictions that could arise if each severity score of interest (e.g. oSCORAD and EASI with overlapping severity items) is modelled separately. EczemaPred can thus be applied to develop predictive models for other AD severity scores, such as EASI and POEM,^19^ a self‐assessment tool recommended by HOME to evaluate subjective symptoms.^20^

EczemaPred has some further advantages, especially for clinical use. The Bayesian framework enables us to make probabilistic predictions by explicitly quantifying uncertainties in parameters and predictions. The state‐space models explicitly describe potential and often inevitable errors in the measurement of the severity items. For example, estimation of the body area affected by eczema is subject to a high inter‐rater variability,^21^ potentially even more so when it is self‐assessed as in PO‐SCORAD.^10^ The choice of representative sites may also introduce variability in the measurement of intensity signs. Modelling the measurement processes separately from the latent dynamics of the disease severity items also allows us to deal with missing values efficiently as an absence of measurement, while still inferring the latent dynamics. In a practical application of the model, the posterior distributions obtained in this study could be used as a prior for new patients to ‘pre‐train’ the model, shortening the training phase to only a few measurements. More generally, the number of data points needed to accurately train the model depends on several factors, including the severity score to predict (e.g. SCORAD or oSCORAD), the performance metric to be optimised (e.g. lpd or accuracy), the target performance (e.g. 60% or 90% accuracy), the prediction horizon (e.g. one day or 1 week), the frequency of measurements (e.g. daily or twice‐weekly) and potentially other characteristics of the datasets (e.g. demographics).

Limitations of this study include the subjective assessment of PO‐SCORAD by patients. The reliability of PO‐SCORAD assessment was shown to improve with experience, as patients may need time to learn how to use the PO‐SCORAD instrument properly.^11^ The severity item models may therefore benefit from specifying a time‐varying measurement error. EczemaPred could also be improved by modelling the correlations between the six intensity signs, even though the components of SCORAD (extent, intensity signs and subjective symptoms) are thought to be uncorrelated by design.^7^ For instance, dryness, thickening and scratching may covary as they mainly characterise the chronicity of the disease; and redness, swelling and oozing may covary as they represent acute flares.^7^ Validation of EczemaPred in a real‐world evidence study is also required, as the data used in this article were taken from patients involved in a clinical study in which they may have had a better follow‐up than usual. The data also lacks severity scores from severe AD patients, who may exhibit different severity trajectory patterns.

In summary, this study introduced EczemaPred as a computational framework to predict the patient‐dependent dynamic evolution of AD severity. Patients could benefit from EczemaPred in managing their disease and anticipating their symptoms' change. Notably, EczemaPred could be used to investigate the effects of treatment and environmental factors on the dynamic evolution of AD severity and eczema persistence. For example, the models could be extended to quantify patients' responsiveness to treatment and suggest personalised treatment strategies using Bayesian decision theory. In conjunction with EczemaNet,^22^ a computer‐vision pipeline to detect and assess eczema severity from camera images, an extended version of EczemaPred could serve as a treatment adjustment tool by providing direct feedback to patients on the likely evolution of their severity and suggesting the most appropriate treatments to manage their condition proactively.

## CONFLICT OF INTEREST

Sophie Mery, Alain Delarue, Markéta Saint Aroman and Gwendal Josse are employees of Pierre Fabre Laboratories. Guillem Hurault, Jean François Stalder and Reiko J. Tanaka have no conflicts to disclose.

## AUTHOR CONTRIBUTIONS

**Guillem Hurault**: Conceptualization, data curation, formal analysis, investigation, methodology, software, validation, visualization, writing‐original draft.

**Jean François Stalder**, **Sophie Mery**: Conceptualization, resources, writing‐review & editing.

**Gwendal Josse**: Resources, data curation, writing‐review & editing.

**Alain Delarue**, **Markéta Saint Aroman**: Resources.

**Reiko J. Tanaka**: Conceptualization, funding acquisition, project administration, resources, supervision, validation, writing‐original draft, writing‐review & editing.

## Supporting information

Supporting Information
