# Supplementary material for: EczemaPred: A computational framework for personalised prediction of eczema severity dynamics
Source: Clin Transl Allergy. 2022 Mar 28;12(3):e12140. doi: 10.1002/clt2.12140 (PMC8967258; doi:10.1002/clt2.12140)
Supplement: Supplementary file 1 — Supporting Information [file CLT2-12-e12140-s001.pdf]

EczemaPred: A computational framework for personalised prediction of eczema severity dynamics

Guillem Hurault, Jean François Stalder, Sophie Mery, Alain Delarue, Markéta Saint Aroman, Gwendal Josse and Reiko J. Tanaka

Supplementary Materials

A. EczemaPred models

We use the following notations in this document.

- $y^{(k)}(t)$ is the score of interest of the k-th patient at time t. k is dropped for models not taking the patient-dependence into account.
- M is the maximum value that y can theoretically take, i.e. $y \in [0, M]$.
- $y \sim \mathcal{N}(\mu, \sigma^2)$ denotes that y is normally distributed with mean μ and variance σ^2.
- $y \sim \mathcal{N}^+(0, \sigma^2)$ denotes that y follows a half-normal distribution (normal distribution defined for positive values) of variance σ^2.
- $y \sim \log \mathcal{N}(\mu, \sigma^2)$ denotes that y is log-normal distributed, i.e. $\log(y) \sim \mathcal{N}(\mu, \sigma^2)$.
- $y \sim \text{logit}\, \mathcal{N}(\mu, \sigma^2)$ denotes that y is logit normal distributed, i.e. $\text{logit}(y) \sim \mathcal{N}(\mu, \sigma^2)$.

A.1. Extent models: Binomial Markov chain

The extent model assumes that we can subdivide the body area into 100 patches, each with a probability, $p(y = 1) = p$, of being classified as lesional, and that each patch has fixed transition probabilities between lesional and non-lesional states. The measurement is specified as a binomial distribution to count the number of lesional patches to produce the extent score, $A \sim \mathcal{B}(100, p)$.

A.1.1. Two-state Markov chain models for latent dynamics

We model the transition from non-lesional to lesional or from lesional to nonlesional for any given patch of the skin with a two-state Markov chain characterised by the transition matrix,

$$T = \begin{pmatrix} p_{00} & p_{01} \\ p_{10} & p_{11} \end{pmatrix} = \begin{pmatrix} 1 - p_{01} & p_{01} \\ p_{10} & 1 - p_{10} \end{pmatrix}, \tag{1}$$

where

- p_{10} is the transition probability from lesional to non-lesional states, i.e. the probability that a lesional patch is classified as non-lesional at the next step,

- $p_{11} = 1 - p_{10}$ is the probability that a lesional patch stays lesional and can be interpreted as a measure of eczema persistence, and
- p_{01} is the transition probability from non-lesional to lesional states, i.e. the probability that a non-lesional patch is classified as lesional at the next step, and can be interpreted as a measure of eczema sensitivity to develop symptoms.

The probability, $p(t)$, of a given patch being lesional is computed by

$$p(t+1) = p_{11}\, p(t) + p_{01}\, \big(1 - p(t)\big). \tag{2}$$

The state of a skin patch at time t is denoted by $x_t = \begin{pmatrix} 1 & 0 \end{pmatrix}$ if it is non-lesional and $x_t = \begin{pmatrix} 0 & 1 \end{pmatrix}$ if it is lesional. The predictions of the state at time $t+h$ is given by $x_{t+h} = x_t T^h$ with

$$T^h = \begin{pmatrix} 1-\pi & \pi \\ 1-\pi & \pi \end{pmatrix} + \lambda^h \begin{pmatrix} \pi & -\pi \\ -(1-\pi) & 1-\pi \end{pmatrix}, \tag{3}$$

where $\lambda = 1 - p_{01} - p_{10}$ is one of the eigenvalues of T (the other one is 1) and $\pi = \frac{p_{01}}{p_{01}+p_{10}}$ characterises the steady state (limiting) distribution, $x_\infty = \begin{pmatrix} 1-\pi & \pi \end{pmatrix}$, to which the Markov chain converges if the prediction horizon, h, is long enough. The value of λ indicates the mobility of the Markov chain, i.e. how fast it converges to the steady state distribution, with $|\lambda| \to 0$ indicating faster convergence.

We assume that the probabilities, p_{01} and p_{10}, are patient-dependent and that either one or both probabilities are time-dependent, given that the Markov chain converges to the steady state distribution, $\pi = \frac{p_{01}}{p_{01}+p_{10}}$, which is likely to evolve over a long enough time. We further assume that p_{10} ($= 1 - p_{11}$, where p_{11} is a measure of eczema persistence) does not exhibit a strong time-dependence and that p_{01} (a measure of eczema sensitivity to develop symptoms) dynamically changes due to endogenous and exogenous factors such as the skin barrier integrity and environmental stressors, respectively. We therefore parametrise the Markov chain by a time (t)- and patient (k)-dependent $\pi^{(k)}(t)$ and a patient-dependent $p_{10}^{(k)}$, from which we can derive

$$p_{01}^{(k)}(t) = p_{10}^{(k)} \frac{\pi^{(k)}(t)}{1 - \pi^{(k)}(t)}.$$

A.1.2. Priors of binomial Markov chain model

The evolution of the extent, $A^{(k)}(t)$, for the k-th patient at time t is modelled with a binomial measurement model and latent Markov Chain model described above[1]:

$$
\begin{aligned}
A^{(k)}(t) &\sim \mathcal{B}(100, p^{(k)}(t)), && (4)\\
p^{(k)}(t+1) &= p_{11}^{(k)}\, p^{(k)}(t) + p_{01}^{(k)}(t)\, \left(1 - p^{(k)}(t)\right), && (5)\\
p_{11}^{(k)} &= 1 - p_{10}^{(k)}, && (6)\\
p_{01}^{(k)}(t) &= p_{10}^{(k)} \frac{\pi^{(k)}(t)}{1 - \pi^{(k)}(t)}, && (7)\\
\pi^{(k)}(t) &= \frac{\tilde{\pi}^{(k)}(t)}{1 + p_{10}^{(k)}}, && (8)\\
\text{logit}\left(\tilde{\pi}^{(k)}(t+1)\right) &\sim \mathcal{N}\Big(\text{logit}\left(\tilde{\pi}^{(k)}(t)\right), \sigma^2\Big), && (9)
\end{aligned}
$$

with the priors,

$$
\begin{aligned}
\sigma &\sim \mathcal{N}^+\Big(0, \left(0.25 \log(5)\right)^2\Big), && (10)\\
p_{10}^{(k)} &\sim \text{logit}\mathcal{N}(\mu_{10}, \sigma_{10}^2), && (11)\\
\tilde{\pi}^{(k)}(t_0) &\sim \text{logit}\mathcal{N}(-1, 1), && (12)\\
\mu_{10} &\sim \mathcal{N}(0, 1), && (13)\\
\sigma_{10} &\sim \mathcal{N}^+(0, 1.5^2). && (14)
\end{aligned}
$$

The priors are chosen to be weakly informative and translate to reasonable prior predictive distributions.

- The prior on σ translates to an odd ratio increment for π of at most 5. That is, $\pi(t) = 0.1$ ($OR(t) = 1/9$) evolves up to $\pi(t+1) = 0.36$ ($OR(t+1) = 5OR(t) \approx 0.56$), which could be considered as an unusually important change.
- The prior for the initial condition, $\tilde{\pi}^k(t_0)$, at t_0 of the first data point is set to be slightly skewed toward 0, as high values of A are very unlikely.
- The priors on μ_{10} and σ_{10} translate to an approximately uniform prior on $p_{10}^{(k)}$.

A.2. Intensity signs models: Ordered logistic random walk

We assume that measurements of the intensity signs are generated from an ordered logistic distribution, which is appropriate to describe ordinal random variables with only a few categories. An ordered logistic distribution is a logistic distribution with a location parameter (latent score), $y_{\text{lat}}^{(k)}(t)$,

[1] The model equations include $\tilde{\pi}^{(k)}(t)$ since the upper bound of $\pi^{(k)}(t)$ conditioned on $p_{10}^{(k)}$ is $\frac{1}{1+p_{10}^{(k)}}$, where p_{01} is between 0 and 1.

that is integrated between M cut-offs, $\boldsymbol{c}$ ($c_0 = 0$[2] $< c_1 < \ldots < c_{M-1}$),

$$
\begin{aligned}
& y^{(k)}(t) \sim \text{OrderedLogistic}\left(y_{\text{lat}}^{(k)}(t), \boldsymbol{c}\right) \\
\iff P\big(y^{(k)}(t) = i\big) = & \begin{cases} 1 - \text{logit}^{-1}\left(y_{\text{lat}}^{(k)}(t) - c_0\right) \text{ if } i = 0, \\ \text{logit}^{-1}\left(y_{\text{lat}}^{(k)}(t) - c_{i-1}\right) - \text{logit}^{-1}\left(y_{\text{lat}}^{(k)}(t) - c_i\right) \text{ for } 0 < i < M-1, \\ \text{logit}^{-1}\left(y_{\text{lat}}^{(k)}(t) - c_{M-1}\right) \text{ if } i = M. \end{cases}
\end{aligned}
\tag{15}
$$

The scale y_{lat} depends on $\boldsymbol{c}$, and is approximately $c_{M-1} - c_0 = c_{M-1}$, so we choose to express priors and the latent dynamic with $\tilde{y}_{\text{lat}}^{(k)}(t) = y_{\text{lat}}^{(k)}(t) * c_{M-1}$, a normalised version of y_{lat}.

We assume that $\tilde{y}_{\text{lat}}^{(k)}(t)$ follows a Gaussian random walk of variance σ^2,

$$
\tilde{y}_{\text{lat}}^{(k)}(t+1) \sim \mathcal{N}\big(\tilde{y}_{\text{lat}}^{(k)}(t), \sigma^2\big). \tag{16}
$$

and use the priors:

$$
\begin{aligned}
\delta &\sim \mathcal{N}^+\big(0, (2\pi/\sqrt{3})^2\big), && (17) \\
\tilde{y}_{\text{lat}}^{(k)}(t_0) &\sim \mathcal{N}(\mu_0, \sigma_0^2), && (18) \\
\mu_0 &\sim \mathcal{N}\big(0.5, 0.25^2\big), && (19) \\
\sigma_0 &\sim \mathcal{N}^+\big(0, 0.125^2\big), && (20) \\
\sigma &\sim \mathcal{N}^+\big(0, 0.1^2\big), && (21)
\end{aligned}
$$

where $\boldsymbol{\delta}$ are the differences between consecutive cutpoints, i.e. $\delta_i = c_{i+1} - c_i$ for $i \in [0, M-1]$.

The priors are chosen to be weakly informative and translate to reasonable prior predictive distributions.

- The prior on δ translates to values less than the width of the standard logistic distribution[3], allowing the possibility of $P\big(y^{(k)}(t) = i\big) \approx 1$.
- The priors on μ_0 and σ_0 translate to an approximately uniform prior for the initial latent score, $y_{\text{lat}}^{(k)}(t_0)$ within the range of y_{lat}.
- The prior on σ assumes it is possible to go from a state where $y = 0$ is the most likely outcome, to a state where $y = M$ is the mostly likely outcome, in two transitions.

[2] We set $c_0 = 0$ without loss of generality since the ordered logistic distribution model is invariant by translation (adding λ to y_{lat} and $\boldsymbol{c}$ does not change the distribution).

[3] The standard deviation of the standard logistic distribution is $\pi/\sqrt{3}$

A.3. Subjective symptoms models: Binomial random walk

The latent score, $y_{\text{lat}}^{(k)}(t)$, for subjective symptoms (discrete variables with a large number categories) is assumed to follow a random walk on the logit scale, and the measurement is modelled by a binomial distribution whose success parameter, p, is allowed to vary with time,

$$y^{(k)}(t) \sim \mathcal{B}(M, p^{(k)}(t)), \tag{22}$$

$$\operatorname{logit}\left(p^{(k)}(t+1)\right) \sim \mathcal{N}\Big(\operatorname{logit}\left(p^{(k)}(t)\right), \sigma^2\Big), \tag{23}$$

with the priors,

$$\sigma \sim \mathcal{N}^+\Big(0, \left(0.25\log(5)\right)^2\Big), \tag{24}$$

$$p^{(k)}(t_0) \sim \operatorname{logit}\mathcal{N}(\mu_0, \sigma_0^2), \tag{25}$$

$$\mu_0 \sim \mathcal{N}(0,1), \tag{26}$$

$$\sigma_0 \sim \mathcal{N}^+(0, 1.5^2). \tag{27}$$

The priors are chosen to be weakly informative and translate to reasonable prior predictive distributions.

- The prior on σ translates to an odd ratio increment for p of at most 5. For instance, if $p(t) = 0.1$ ($OR(t) = 1/9 \approx 0.11$), then the prior assumes it is unusual, but not impossible, that the next value is $p(t+1) = 0.36$ ($OR(t+1) = 5OR(t) = 5/9 \approx 0.56$).
- The priors on μ_0 and σ_0 have a reasonable range and translate to a marginal prior for $p^{(k)}(t_0)$ that is approximately uniform.

B. Performance metrics

This study used probabilistic models whose prediction is a distribution. Severity items are considered to be discrete and their predictions are described by probability mass functions, while PO-(o)SCORAD is treated as a continuous variable[4] and its predictions are described by probability density functions.

Let $p(y|\theta)$ denote the predictive distribution of a score, y, given the parameters, θ. The expected predictive distribution, marginalised over the posterior distribution, $p_{\text{post}}(\theta)$, is

$$p(y) = \int p(y|\theta) p_{\text{post}}(\theta) d\theta. \tag{28}$$

B.1. Log predictive density (lpd)

We compute the logarithmic scoring rule, aka the log predictive density, of a single data-point, y_n, as

$$\mathit{lpd}(y_n) = \log p(y_n). \tag{29}$$

The lpd's of single data-points are then averaged to produce the lpd for a set of predictions. The lpd is defined between $-\infty$ and ∞ (between $-\infty$ and 0 when using probability mass functions) where a larger lpd indicates a better model.

B.2. Accuracy

We also compute an accuracy metric to facilitate the interpretation of the performance of PO-(o)SCORAD predictions. Since the accuracy is not a proper scoring rule, it is used only for model interpretation but not for model selection.

We define the accuracy, Acc_n^d, for a single data-point, y_n, and a fixed threshold, d, by the probability that the absolute error $|\epsilon_n| = |\hat{y}_n - y_n|$ is less than d, where $\hat{y}_n$ denotes the random variable whose distribution is the predictive distribution with density $p(y)$,

$$\begin{aligned} Acc_n^d &= P(|\hat{y}_n - y_n| < d) \\ &= \int_{y_n - d}^{y_n + d} p(y) dy. \end{aligned} \tag{30}$$

Here, the threshold d represents a maximum acceptable error, and should ideally be a Minimal Important Difference (MID) or a Minimal Detectable Change (MDC). In this study, we arbitrarily chose $d = 5$, which is smaller than published estimates of the MID of 8.7 for SCORAD and 8.2 for oSCORAD [1]. We preferred to take a smaller values than the published estimates of MID, especially because the uncertainty around these estimates were not reported in [1].

[4]Otherwise, PO-SOCRAD is a discrete ordinal variables with 1031 categories

The accuracy of single data-points are then averaged to produce the accuracy for a set of predictions. The accuracy is defined in [0, 1]. However, it should be noted that the maximum accuracy could be less than 100% if $d < \mathit{MDC}$. Conversely, if a 100% accuracy is achieved for a certain value, $\hat{d}$, it suggests that $\mathit{MDC} \leq \hat{d}$.

B.3. Learning curves

We obtained a learning curve by averaging the lpd (resp. the accuracy) at each training/testing iteration. The average is taken over all predictions in the test set. This average is mostly equivalent to the average of the predictions across patients. However, the population of patients that we averaged across is not always the same for different training iterations due to missing observations. For instance, it is possible that we average across patients 1 and 2 to compute the lpd at the i-th iteration and across patients 1 and 3 at the $(i + 1)$-th iteration. This could cause an issue if the model is consistently better at predicting the patient 2 than the patients 1 and 3, as the performance at the i-th iteration could be higher than that at the $(i+1)$-th iteration even though the model is learning from the i-th to the $i + 1$-th iterations. This phenomenon is known as Simpson's paradox which happens because we fail to consider the patient IDs as a confounding factor [2]. Another confounding factor is the horizon of predictions, which may differ on average from one iteration to the next.

To address this potential issues, we proposed a meta-model to estimate the mean lpd (resp. the mean accuracy) of the test dataset. We fitted a model to explain the lpd as a function of the number of observations in the training set, the prediction horizon and the patient IDs and use the mean fit as the lpd (resp. accuracy) estimate. We used a Generative Additive Model (GAM) with cubic splines,

$$\mathit{lpd} \sim s\big(N(i)\big) + t + (1 \,|\, \mathit{Patient}), \tag{31}$$

to achieve a flexible fit to the evolution of the lpd while avoiding overfitting. In (31),

- $s\big(N(i)\big)$ corresponds to a cubic spline on the number of observations, $N(i)$, in the training set at the i-th iteration. $N(i)$ is proportional to i except for late iterations, where a significant fraction of patients have dropped out of the study.
- A cubic spline on x, $s(x)$, can be written as $\beta_1 b_1(x) + \beta_2 b_2(x) + ...$, a linear combination of piecewise cubic polynomial basis functions of x, $b_j(x)$, and coefficients β_j.
- t corresponds to the prediction horizon. For simplicity, we assume that the decrease in performance is linear and does not interact with i.
- $(1 \,|\, \mathit{Patient})$ represents a mixed effect on the intercept for different patients.

The model was fitted using the gamm4 package in R.

C. Reference models

C.1. Uniform forecast models

A uniform forecast assumes that observations follow a discrete/continuous uniform distribution, $y(t+1) \sim \mathcal{U}(0, M)$. It means $P\big(y(t+1) = i\big) = \frac{1}{M+1}$ for all $i \in [0, M]$ for discrete variables.

C.2. Historical forecast models

A historical forecast model makes heuristic forecasts based on the prevalence of past observations. For instance, if 10% of observations in the training set are 0, then $P\big(y(t+1) = 0\big) = 0.1$ for the discrete case. For the continuous case, it is not possible to compute a probability table from past observations. Therefore, the lpd was computed using kernel density estimate and the accuracy is computed as if the training set was Monte Carlo samples.

It is worth noting that a historical forecast model may require a lot of data before having stable estimates, especially if the number of categories (in the discrete case) is large. As a result, the historical forecast model is patient-independent, as a patient's time-series are too short to estimate a patient-dependent historical forecast accurately.

C.3. Random walk models

A random walk model provides a flat forecast, i.e. a forecast centered on the last observation with the uncertainty quantified by a variance parameter σ^2, and is described by

$$y(t+1) \sim \mathcal{N}\big(y(t), \sigma^2\big), \tag{32}$$

with the prior for the variance σ^2 defined by

$$\sigma \sim \mathcal{N}^+\big(0, (0.1M)^2\big). \tag{33}$$

The scale of the prior was set to be 10% of the range, M, of the score. That is, we expect σ to be approximately at most $0.2M$, which further translates in a width of the 95% prediction interval to be $0.8M$ (i.e. almost the range of the score, considering the approximations).

The random walk model was trained in a semi-supervised setting where missing values were treated as parameters to be inferred by the model. The model is considered to be "naive" in the sense it uses a non-truncated distribution and implicitly treats y as continuous (since the Gaussian is defined for continuous variables) and thus performs similarly to standard off-the-shelf implementations. However, the predictive distributions are truncated for proper evaluation, so that the predictive distribution integrates to one over the support of the score. In the case of discrete variables, predictions were also discretised (rounded to the nearest integer).

C.4. Markov chain models

When the number of discrete categories is small (e.g. for intensity signs), a Markov chain model may be more appropriate than a random walk model in order to provide a somewhat flat forecast.

This model assumes that the evolution of y is described by a Markov chain with $M + 1$ states and $P\big(y(t+1) = j \,|\, y(t) = i\big) = p_{i,j}$. More generally, $P\big(y(t+h) = j \,|\, y(t) = i\big) = (T^h)_{i,j}$, for a h-steps-ahead transition, where T is the transition matrix, $(T)_{i,j} = p_{i,j}$. As a baseline model, the transition probabilities, $p_{i,j}$, are assumed to be patient- and time-independent. For the vector, $\boldsymbol{p_i}$, representing the transition probability distribution from state i, we assume an uninformative uniform prior over $\boldsymbol{p_i}$ using a symmetric Dirichlet distribution,

$$\boldsymbol{p_i} \sim \text{Dirichlet}(1, ..., 1). \tag{34}$$

C.5. Exponential smoothing models

An exponential smoothing model corresponds to an exponential smoothing of the data and a flat forecast, and is described by

$$l(t) \quad = \alpha y(t) + (1 - \alpha) l(t-1), \tag{35}$$

$$y(t+1) \sim \mathcal{N}\big(l(t), \sigma^2\big), \tag{36}$$

where l represents the smoothed values (the level) and α is the smoothing factor, which can be related to the time constant τ of the process and the delay $\Delta T = 1$ day between two observations by

$$\alpha = 1 - e^{-\frac{\Delta T}{\tau}} \iff \tau = -\frac{\Delta T}{\log(1 - \alpha)}. \tag{37}$$

We assume the priors,

$$\tau \sim \log\mathcal{N}\big(0.5\log(10), (0.75\log(10))^2\big), \tag{38}$$

$$\sigma \sim \mathcal{N}^+\big(0, (0.1M)^2\big), \tag{39}$$

where τ follows a log-normal prior to span several orders of magnitude between 10^{-1} and 10^2 days.

C.6. Autoregressive models

An autoregressive model is an extension of the "random walk" model with an autocorrelation coefficient, α, and an intercept, $b = (1 - \alpha) y_\infty$, where y_∞ is the expected value of the series (we assume stationarity),

$$y(t+1) \sim \mathcal{N}\big(\alpha y(t) + b, \sigma^2\big), \tag{40}$$

with the priors,

$$\begin{aligned}
\alpha &\sim \mathcal{U}(0,1), && (41)\\
y_\infty &\sim \mathcal{N}\big(0.5M, (0.25M)^2\big), && (42)\\
\sigma &\sim \mathcal{N}^+\big(0, (0.1M)^2\big). && (43)
\end{aligned}$$

The prior on y_∞ results in a 95% CI being $[0, M]$ (the range of the score). The prior on σ has the same rational as for the random walk model.

C.7. Mixed autoregressive models

A mixed autoregressive model is an extension of the "Autoregressive model" by assuming patient-dependence for the autocorrelation, $\alpha^{(k)}$, and the intercept, $b^{(k)} = (1 - \alpha^{(k)})y_\infty^{(k)}$, described by

$$y^{(k)}(t+1) \sim \mathcal{N}\big(\alpha^{(k)}y^{(k)}(t) + b^{(k)}, \sigma^2\big), \tag{44}$$

with the priors,

$$\begin{aligned}
\alpha^{(k)} &\sim \operatorname{logit}\mathcal{N}(\mu_\alpha, \sigma_\alpha^2), && (45)\\
\mu_\alpha &\sim \mathcal{N}(0,1), && (46)\\
\sigma_\alpha &\sim \mathcal{N}^+(0, 1.5^2), && (47)\\
y_\infty^{(k)} &\sim \mathcal{N}(\mu_\infty, \sigma_\infty^2), && (48)\\
\mu_\infty &\sim \mathcal{N}\big(0.5M, (0.25M)^2\big), && (49)\\
\sigma_\infty &\sim \mathcal{N}^+\big(0, (0.125M)^2\big), && (50)\\
\sigma &\sim \mathcal{N}^+\big(0, (0.1M)^2\big). && (51)
\end{aligned}$$

The priors are chosen to be weakly informative and translate to reasonable prior predictive distributions.

- The priors on μ_α and σ_α have reasonable ranges and translate to a marginal prior for $\alpha^{(k)}$ that is approximately uniform.
- The prior for μ_∞ spans the range [0, M] in which y is defined.
- The prior for σ_∞ implies that the range of the distribution of y_∞ is at most M.

References

[1] M. E. Schram, P. I. Spuls, M. M. G. Leeflang, R. Lindeboom, J. D. Bos, and J. Schmitt, "EASI, (objective) SCORAD and POEM for atopic eczema: Responsiveness and minimal clinically important difference," *Allergy: European Journal of Allergy and Clinical Immunology*, vol. 67, no. 1, pp. 99–106, 2012.

[2] T. Gneiting and A. E. Raftery, "Strictly proper scoring rules, prediction, and estimation," *Journal of the American Statistical Association*, vol. 102, pp. 359–378, 3 2007.

Supplementary Figures

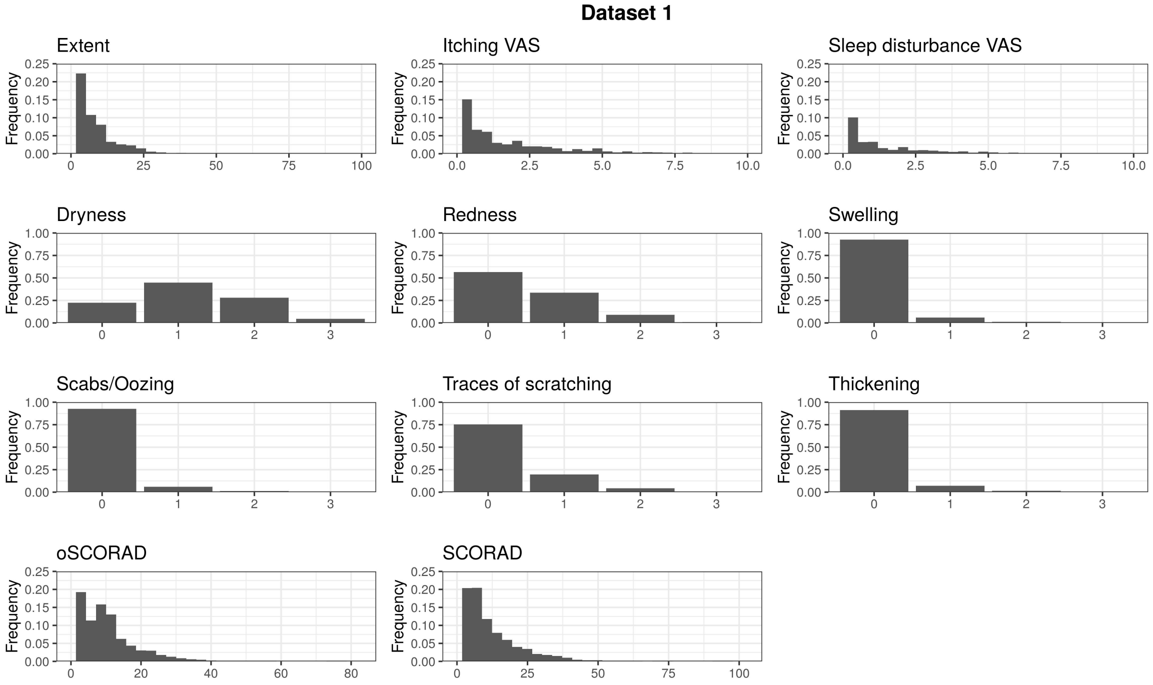

Figure S1: Distribution of the nine severity items and (o)PO-SCORAD in dataset 1.

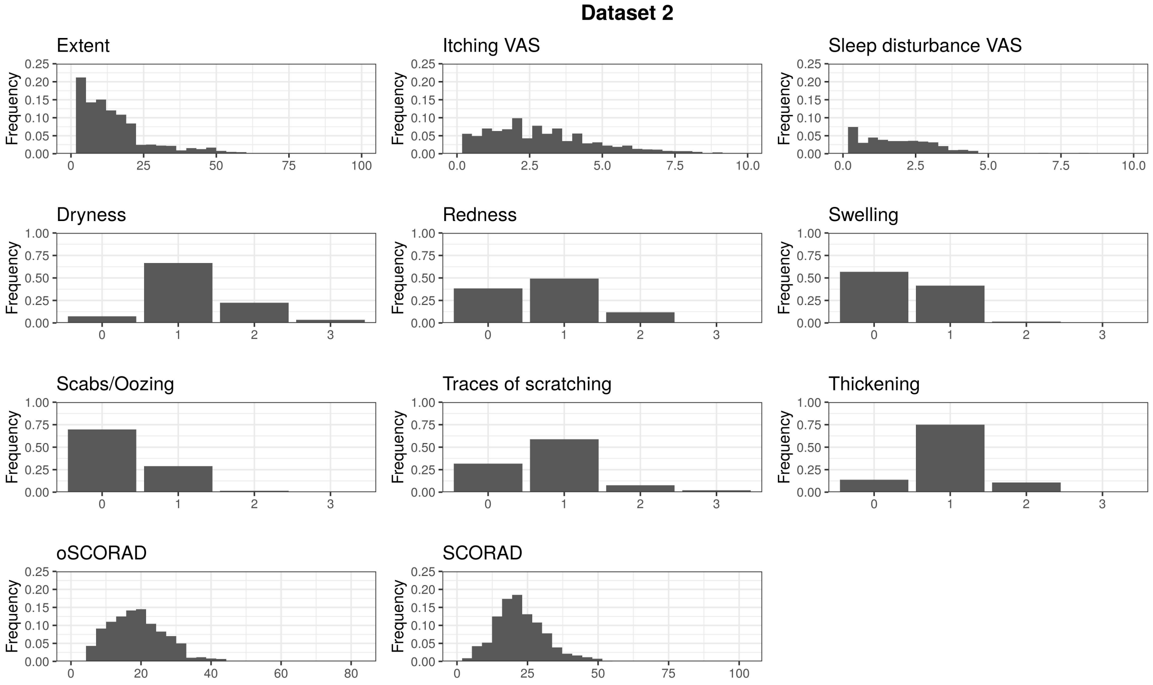

Figure S2: Distribution of the nine severity items and (o)PO-SCORAD in dataset 2.

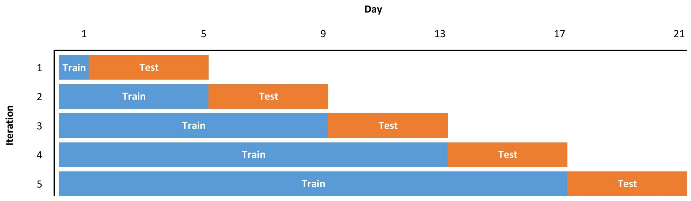

Figure S3: An overview of the forward chaining setting used in this paper. We first trained the models on the first day's data of a patient and tested them using their data over the next four days, then trained the models on the first five days' data and tested them on the next four days' data, etc.

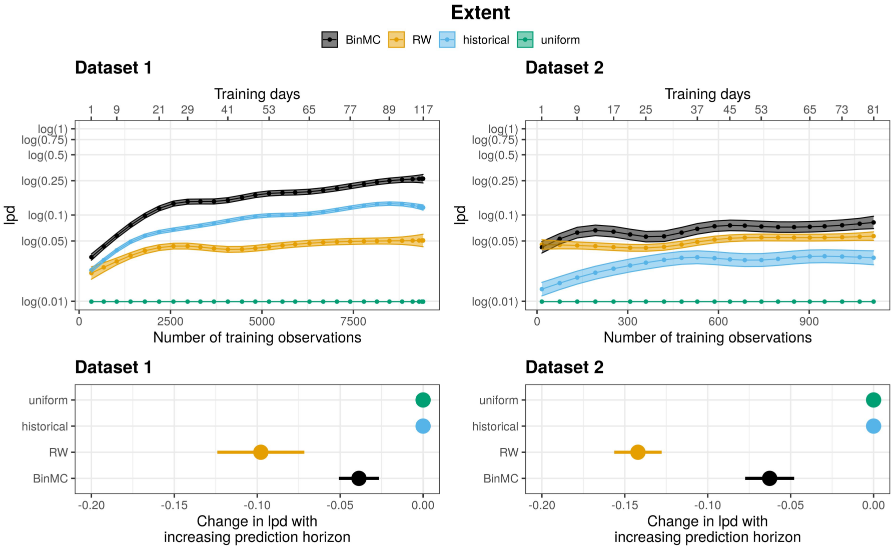

Figure S4: Predictive performance of the Extent model with datasets 1 (left) and 2 (right), evaluated by lpd ($\pm$ SE, the higher the better). Top: Learning curves for 4-days-ahead predictions as a function of the number of training days (top axis) and the number of training observations (bottom axis). Bottom: Change in lpd as the prediction horizon is increased by a day.

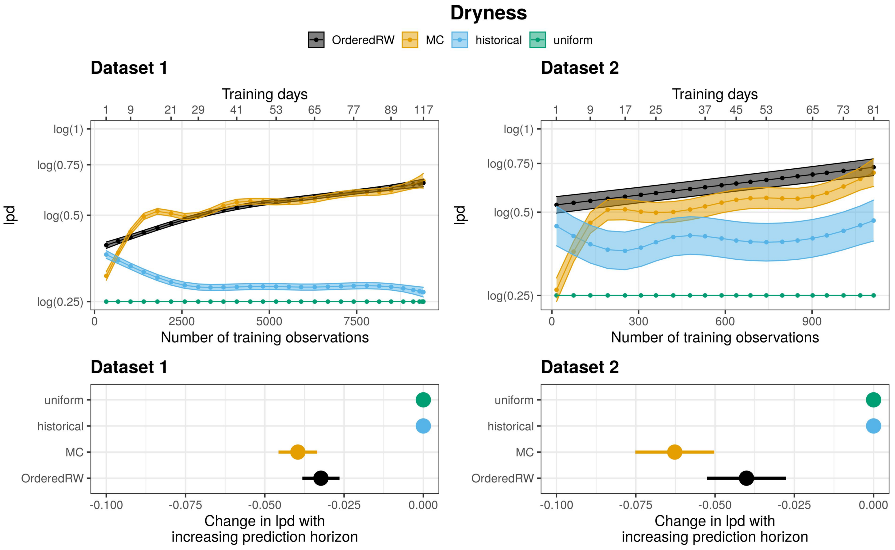

Figure S5: Predictive performance of the Dryness model with datasets 1 (left) and 2 (right), evaluated by lpd ($\pm$ SE, the higher the better). Top: Learning curves for 4-days-ahead predictions as a function of the number of training days (top axis) and the number of training observations (bottom axis). Bottom: Change in lpd as the prediction horizon is increased by a day.

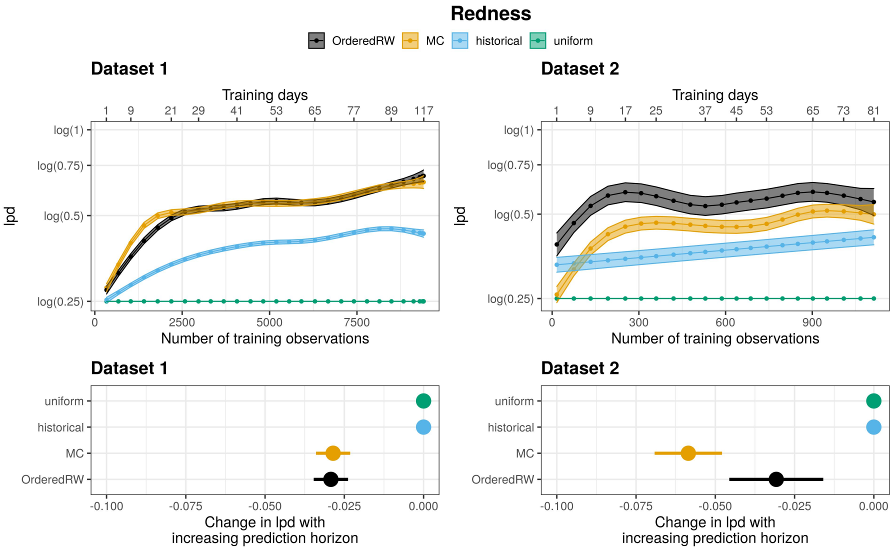

Figure S6: Predictive performance of the Redness model with datasets 1 (left) and 2 (right), evaluated by lpd ($\pm$ SE, the higher the better). Top: Learning curves for 4-days-ahead predictions as a function of the number of training days (top axis) and the number of training observations (bottom axis). Bottom: Change in lpd as the prediction horizon is increased by a day.

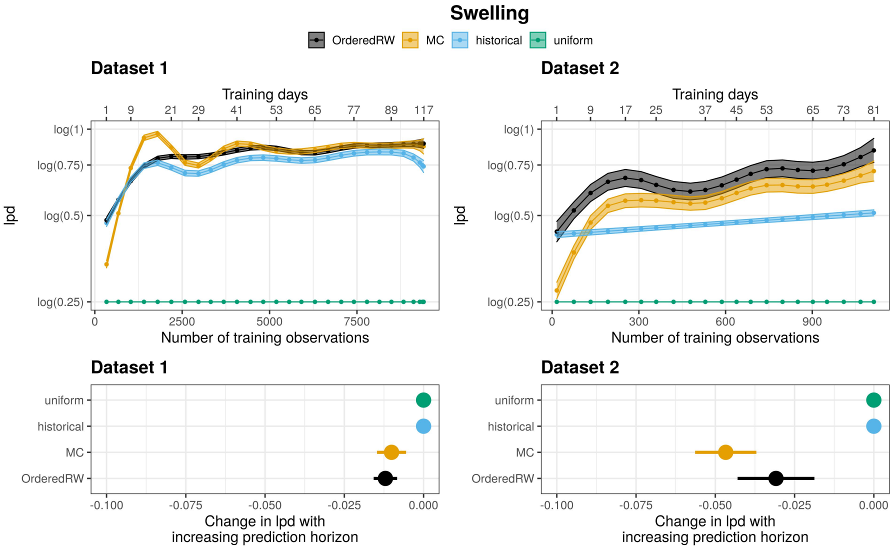

Figure S7: Predictive performance of the Swelling model with datasets 1 (left) and 2 (right), evaluated by lpd (± SE, the higher the better). Top: Learning curves for 4-days-ahead predictions as a function of the number of training days (top axis) and the number of training observations (bottom axis). Bottom: Change in lpd as the prediction horizon is increased by a day.

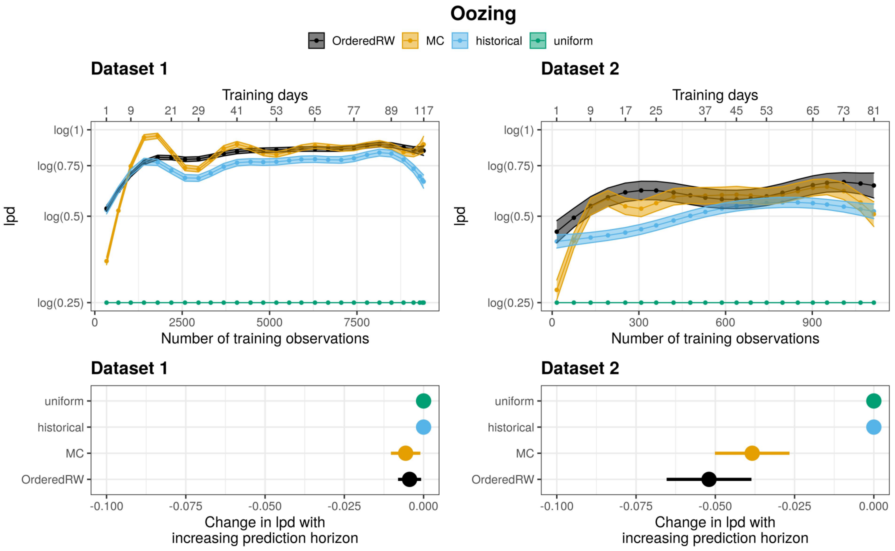

Figure S8: Predictive performance of the Oozing model with datasets 1 (left) and 2 (right), evaluated by lpd (± SE, the higher the better). Top: Learning curves for 4-days-ahead predictions as a function of the number of training days (top axis) and the number of training observations (bottom axis). Bottom: Change in lpd as the prediction horizon is increased by a day.

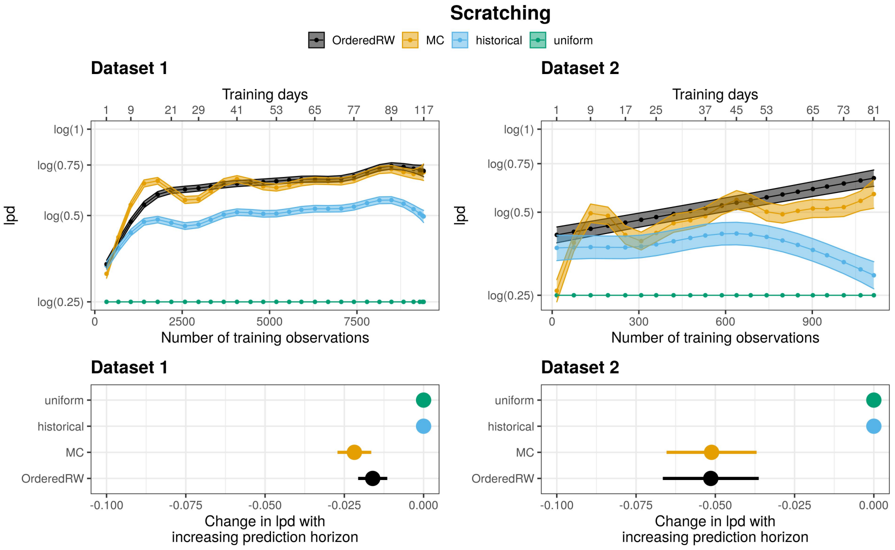

Figure S9: Predictive performance of the Scratching model with datasets 1 (left) and 2 (right), evaluated by lpd (± SE, the higher the better). Top: Learning curves for 4-days-ahead predictions as a function of the number of training days (top axis) and the number of training observations (bottom axis). Bottom: Change in lpd as the prediction horizon is increased by a day.

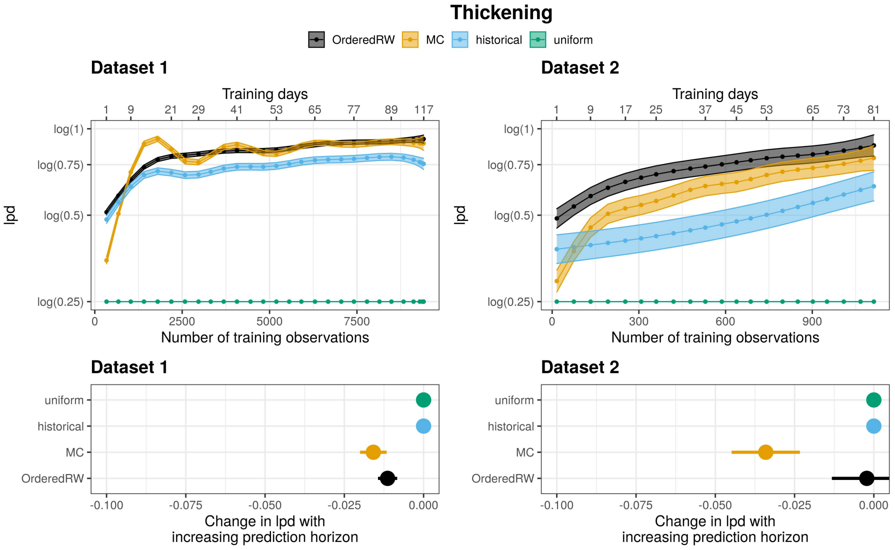

Figure S10: Predictive performance of the Thickening model with datasets 1 (left) and 2 (right), evaluated by lpd (± SE, the higher the better). Top: Learning curves for 4-days-ahead predictions as a function of the number of training days (top axis) and the number of training observations (bottom axis). Bottom: Change in lpd as the prediction horizon is increased by a day.

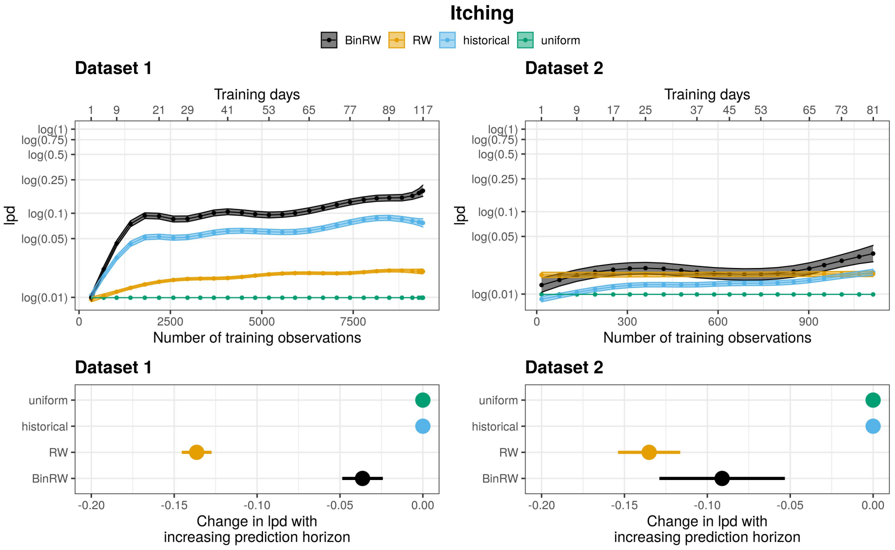

Figure S11: Predictive performance of the Itching model with datasets 1 (left) and 2 (right), evaluated by lpd (± SE, the higher the better). Top: Learning curves for 4-days-ahead predictions as a function of the number of training days (top axis) and the number of training observations (bottom axis). Bottom: Change in lpd as the prediction horizon is increased by a day.

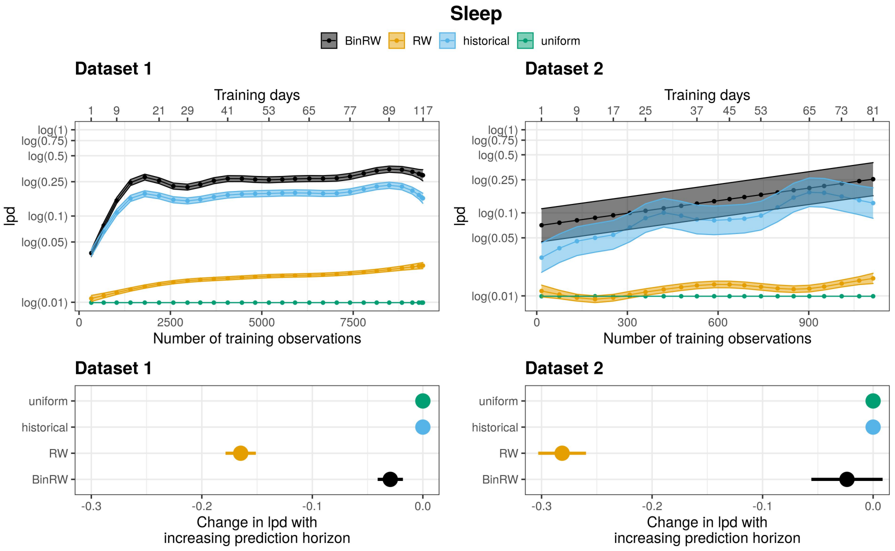

Figure S12: Predictive performance of the Sleep loss model with datasets 1 (left) and 2 (right), evaluated by lpd (± SE, the higher the better). Top: Learning curves for 4-days-ahead predictions as a function of the number of training days (top axis) and the number of training observations (bottom axis). Bottom: Change in lpd as the prediction horizon is increased by a day.

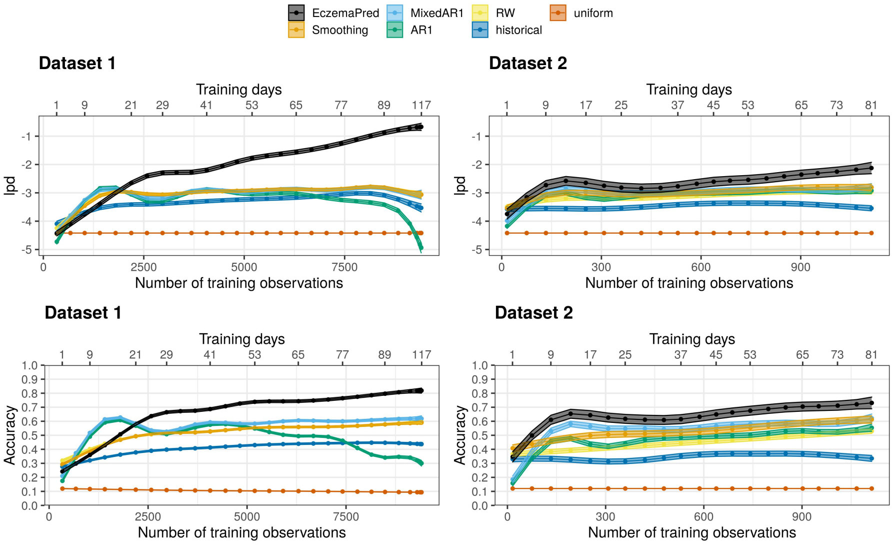

Figure S13: Learning curves of models predicting PO-oSCORAD, measured by lpd (top) and accuracy (bottom) as a function of the number of training observations (and the number of training days), for datasets 1 (left) and 2 (right). EczemaPred model performs better than the reference models.

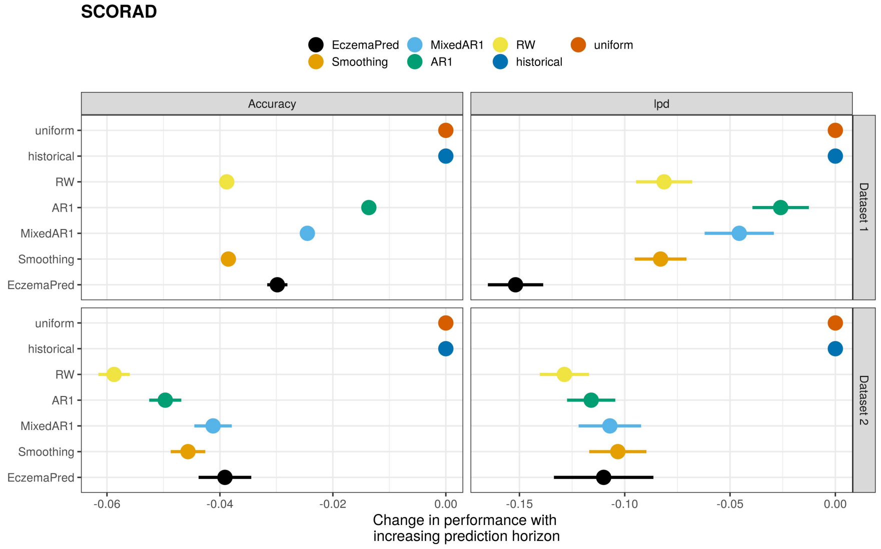

Figure S14: PO-SCORAD predictive performance change as the prediction horizon is increased by one day, measured by Accuracy (left) and lpd (right), for datasets 1 (top) and 2 (bottom). The predictive performance for PO-SCORAD decreased with an increase in prediction horizon for all models.

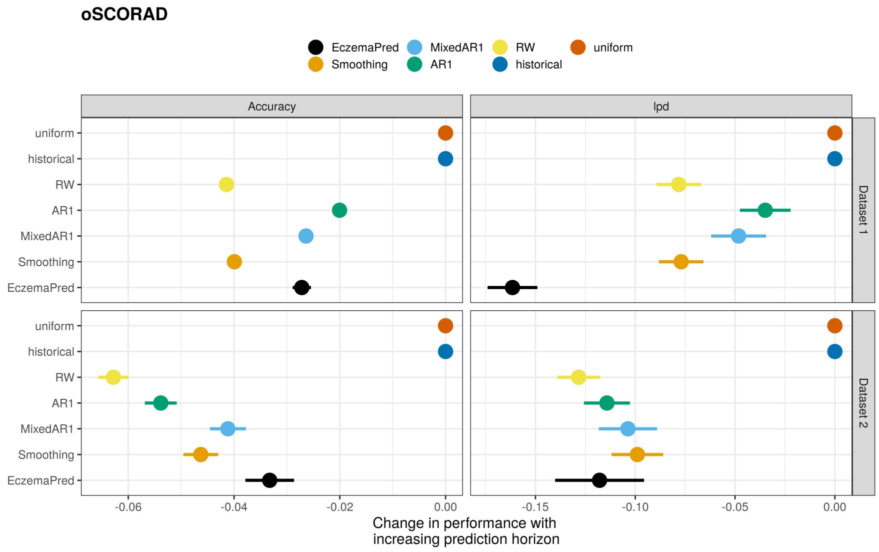

Figure S15: PO-oSCORAD predictive performance change as the prediction horizon is increased by one day, measured by Accuracy (left) and lpd (right), for datasets 1 (top) and 2 (bottom).
